# Protective effect of apigenin magnesium complex on H_2_O_2_-induced oxidative stress and inflammatory responses in rat hepatic stellate cells

**DOI:** 10.1080/13880209.2020.1772840

**Published:** 2020-06-16

**Authors:** Xuwang Pan, Yidan Shao, Fugen Wang, Zhaobin Cai, Shourong Liu, Jianjun Xi, Ruoyu He, Yanmei Zhao, Rangxiao Zhuang

**Affiliations:** a Department of Pharmaceutical Preparation, Hangzhou Xixi Hospital, Hangzhou, Zhejiang, China; b Department of Liver Disease, Hangzhou Xixi Hospital, Hangzhou, Zhejiang, China

**Keywords:** MTT, redox system, proinflammatory cytokines, real-time PCR

## Abstract

**Context:**

Apigenin displays antioxidant and anti-inflammatory effects. However, effects of apigenin magnesium (AM) complex on these aspects remain unknown.

**Objective:**

This study investigated the effects of AM complex on oxidative stress and inflammatory responses in hydrogen peroxide (H_2_O_2_)-induced rat hepatic stellate cells (HSCs).

**Materials and methods:**

The antioxidant and anti-inflammatory effects of AM complex at concentrations of 0.625, 1.25, and 2.5 mg/mL were evaluated, comparing to HSCs treated by H_2_O_2_ alone. Cell viability, reactive oxygen species (ROS), the activity of superoxide dismutase (SOD), glutathione (GSH), malondialdehyde (MDA), nitric oxide (NO), interleukin 6 (IL-6), and nuclear factor-kappa B (NF-κB) levels were measured. Moreover, cell apoptosis, mRNA expression levels of transforming growth factor-β (TGF-β), NF-κB, and inducible nitric oxide synthase (iNOS) were assessed.

**Results:**

AM complex significantly inhibited oxidative stress and inflammatory response at concentrations of 0.625, 1.25, and 2.5 mg/mL (IC_50_ = 1.679 mg/mL). AM complex elevated the survival rate of H_2_O_2_-treated HSCs and had no toxic effects on HSCs. AM complex also promoted SOD activity and GSH levels but suppressed ROS, MDA, and NO levels. Additionally, AM complex decreased IL-6 and NF-κB levels, gene expression of TGF-β, NF-κB, and iNOS, as well as induced apoptosis of HSCs.

**Discussion and conclusions:**

Data indicated that AM complex mitigated oxidative stress and inflammatory responses on H_2_O_2_-treated HSCs, suggesting that AM complex is a possible candidate for anti-hepatic diseases. Additional efforts, both *in vivo* and in humans, are required to assess of AM complex as a potential therapeutic drug in liver diseases.

## Introduction

Hepatic diseases, ranging from early steatosis to severe hepatitis, fibrosis, cirrhosis, and hepatocellular carcinoma (HCC), have a high incidence worldwide (Li et al. 2016). Hepatic disease affects more than 10% of the world population and its deadly end-stage usually occurs after cirrhosis and liver cancer. Various aetiologies rank the disease as the fourth or fifth leading cause of death worldwide. Hepatic diseases may be caused by various risk factors, such as obesity, virus, alcohol, drugs, and other toxins (Obert et al. 2015). Since the liver is the central organ for excreting toxins and metabolic nutrients, it is more vulnerable to oxidative stress and inflammation produced from toxins and metabolites in the body (Li et al. 2015). Substantial animal studies and clinical trials have shown that persistent oxidative stress and inflammation in the liver are crucial in the occurrence and development of hepatic illness, regardless of the aetiology. Both persistent oxidative stress and inflammation are regarded as vital factors in the pathogenesis of acute and chronic liver diseases. Oxidative stress refers to cellular or molecular damage caused by reactive oxygen species (ROS), due to the imbalance between ROS production and antioxidant defense responses. Oxidative stress causes hepatic damage through altering biological molecules, such as DNA, proteins, and lipids, and modulating biological pathways associated with gene transcription, protein expression, cell apoptosis, and hepatic stellate cell (HSC) activation (Li et al. 2015). Inflammation is an important part of the immune response, manifested in the fact that inflammatory cells penetrate mainly to the liver to combat pathogen invasion. However, once stimuli persist or are overwhelming, they cause cell damage and lipid accumulation, increasing the risk of severe liver diseases such as steatohepatitis, fibrosis and cancer (Seki and Schwabe 2015). A better understanding of the potential roles of oxidative stress and inflammation in hepatic disease pathogenesis may provide novel opportunities for therapeutic intervention to prevent progression to advanced disease.

**Figure 1.**
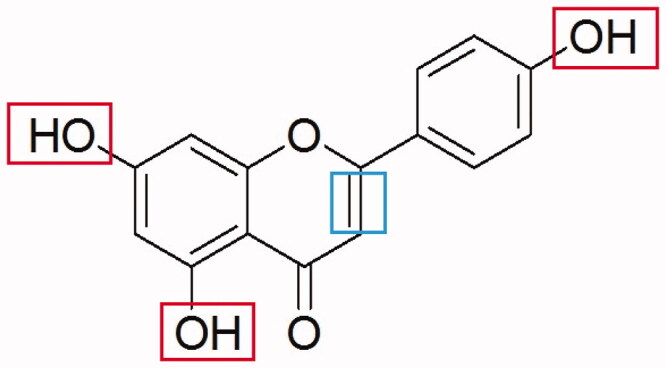
Structural formula of apigenin. The three rectangles indicate the three hydroxyl groups. The square indicates the double bonds at C2 and C3.

Apigenin (4′, 5, 7-trihydroxyflavone), a natural plant flavone, is found in many plants, such as herbs, spices, citrus fruit, vegetables, and other foods (Sung et al. 2016; Liu et al. 2017). Experimental studies demonstrated that apigenin has some pharmacological activity, such as antioxidant (Zhou et al. 2017; Wang et al. 2018), anti-inflammatory (Kilani-Jaziri et al. 2016; Patil et al. 2016), and anticancer (Salmani et al. 2017; Madunic et al. 2018) activities. Our previous studies also have shown that apigenin has a protective effect on hepatic fibrosis induced by CCl_4_ and non-alcoholic fatty liver disease (NAFLD) induced by a high fat diet (Xi et al. 2014; Shao et al. 2015; Shi et al. 2015; Zhuang et al. 2016; Pan et al. 2017). The mechanism may be related to lipid peroxide inhibition, the clearance of free radicals, and the reduction of inflammatory factors (Xi et al. 2014; Shao et al. 2015; Zhuang et al. 2016). Research indicates that the unique pharmacological and biological effects of apigenin are mainly determined by three hydroxyl groups at its 4′, 5, 7 position and double bond at C2 and C3 (Figure 1). As a natural product with a high effect and low toxicity, apigenin has a great potential for development. However, due to poor hydrophilicity and poor lipophilicity, the clinical application of apigenin is greatly limited. Therefore, in our previous experiments, apigenin and magnesium carbonate in alkaline solution were used to form an apigenin magnesium (AM) complex to improve its solubility (Pan et al. 2014). Considering its partial acidity, strong super delocalization, large π bond conjugation systems of benzopyrone, and the strong coordination ability of the oxygen atoms, the spatial structure of apigenin is conducive to complex formation (Badea et al. 2011; Lu et al. 2011). Magnesium is an essential element in the normal life and metabolism of an organism. Combining apigenin and magnesium ions may form a new complex that improves physical and chemical properties and functions of apigenin. Given the potent antioxidant, free radical scavenging, and anti-inflammatory effects of apigenin, we herein hypothesized that AM complex may have similar or even better activities (Li et al. 2016; Medina et al. 2017).

Hepatic fibrosis is one of the common pathological bases of all chronic hepatic diseases, and the key factor affecting hepatic fibrosis is the activation of HSCs and its transformation into myofibroblasts (Lucey et al. 2013). Many factors, such as inflammatory factors, changes in extracellular matrix, growth factors, and oxidative stress, can stimulate the activation of HSCs (Tsuchida & Friedman 2017; Wang et al. 2017; Cheng et al. 2019; Yan et al. 2019). For these reasons, HSCs were used to establish the oxidative stress cellular model induced by hydrogen peroxide (H_2_O_2_). We used this model to examine the protective effects of AM complex on oxidative stress and inflammatory responses.

## Materials and methods

### Chemical compounds and reagents

AM complex was prepared as previously reported (Pan et al. 2014) and authenticated with UV, IR, and elemental analysis. The main process was as follows: To a solution of apigenin (1.35 mg, 5 mmol) in EtOH (100.0 ml) was added Mg(Ac)_2_ (0.71 mg, 5 mmol) in EtOH (100.0 mL). The mixture was adjusted pH to 9.0 with NaOH (0.05 mol/L), then stirred at 50 °C for 1 h, 1000 rpm. The reaction mixture was cooled to room temperature, the residue was washed with EtOH 50 mL twice, thereafter, washed with acetone/acetic acid, and filtered and concentrated at 50 °C, under reduced pressure to give a target compound (AM complex). *N*-acetylcysteine (NAC), dimethyl sulfoxide (DMSO) and 3-(4,5-dimethylthiazol-2-yl)-2,5-diphenyltetrazolium bromide (MTT) were purchased from Sigma-Aldrich (St. Louis, MO, USA). Dulbecco’s modification of Eagle’s medium (DMEM) was purchased from Hyclone (Logan, UT, USA). Collagenase Type I, Collagenase Type II, and trypsin were purchased from Worthington Biochemical Corporation (Lakewood, NJ, USA). Enzyme-linked immunosorbent assay (ELISA) kits for detecting interleukin 6 (IL-6) and nuclear factor-kappa B (NF-κB) level were purchased from Elabscience (Wuhan, China). Kits for detecting nitric oxide (NO) levels and 0.25% pancreatic enzymes were purchased from Shanghai Biyuntian Biotechnology Co., Ltd (Shanghai, China). Kits for detecting superoxide dismutase (SOD) activity, glutathione (GSH), and malondialdehyde (MDA) content were purchased from Nanjing Jiancheng Bioengineering Institute (Nanjing, China). A Hoechst 33258 stain was purchased from KeyGen Biotech Co., Ltd. (Jiangsu, China). TRIzol reagent was purchased from Thermo Fisher Scientific (Waltham, MA, USA).

### Separation, culture and treatments of primary HSCs

The liver of a male Sprague-Dawley (SD) rat was perfused with enzymes at two steps for the digestion of collagen, hepatic parenchyma cells, and their released DNA, *in situ* perfusion with pre-perfusion fluid, and *ex vivo* perfusion with enzyme-containing perfusion fluid. HSCs were harvested after removing confounding cells by centrifugation (450 *g*, 5 min). Cell viability was determined after trypan blue staining. HSCs were passaged when in a logarithmic growth period after 0.25% trypsin digestion. After amplification, the cell concentration was adjusted to 6 × 10^4^ cells/mL. HSCs were inoculated on a culture plate and cultured in a humidified incubator in an atmosphere of 95% air and 5% CO_2_ at 37 °C. Cells incubated with culture medium alone were used as a negative control. HSCs were pre-treated with various concentrations of AM complex (0.625, 1.25, and 2.5 mg/mL) and NAC (0.82 mg/mL) for 0.5 h, and exposed to H_2_O_2_ (100 μM) for another 48 h. AM complex and NAC were dissolved in DMEM in all experiments. Each experiment was repeated three times.

### Evaluation of toxicity of AM complex in HSCs

HSCs were seeded in 96-well plates (6 × 10^3^/well) and cultured in DMEM with 10% foetal bovine serum (FBS) for 24 h. Cells were treated with AM complex at concentrations of 0, 0.625, 1.25, 2.5, 5.0, and 10 mg/mL for 0.5 h. Then 15 μL of MTT (5 mg/mL) were added to each well and incubated at 37 °C for 4 h. Next, 200 µL of DMSO were added to dissolve the crystals. The spectrophotometric absorbance was measured at 492 nm. The cell survival rate equaled [1−(test_OD value_−blank_OD value_)/(control_OD value_−blank_OD value_)] × 100%.

### Evaluation of AM complex protection of HSCs against oxidative injury

HSCs were seeded in 96-well plates (6 × 10^3^/well) and cultured in DMEM with 10% FBS for 24 h. HSCs were then treated with AM complex/NAC and H_2_O_2_ at indicated concentrations as described above. Cell viability was evaluated using the MTT assay as described above to evaluate AM complex protection against oxidative injury in HSCs.

### ROS assessment

The intracellular ROS was determined using a dihydroethidium (DHE) probe (Shanghai Biyuntian Biotechnology Co., Ltd, Shanghai, China). HSCs were seeded in 96-well plates and cultured in DMEM with 10% FBS for 24 h, and then treated with various concentrations of AM complex/NAC and H_2_O_2_ as described above. Then, the culture medium was removed, and cells were incubated with DHE solutions (10 μM) for 20 min. After that, cells were washed three times to thoroughly remove excess DHE. Micrographs were taken under a fluorescence microscope (Olympus, Tokyo, Japan). The excitation and emission wavelengths were set at 300 and 610 nm, respectively.

### ELISA analysis

HSCs were seeded in 96-well plates and cultured in DMEM with 10% FBS for 24 h. Cells were then treated with AM complex/NAC and H_2_O_2_ at indicated concentrations as described above. The cell supernatant was collected to analyse NO, IL-6, and NF-κB levels. The cells were lysed with cell lysate for 1 h and centrifuged at 13200 *g* for 15 min. Subsequently, the supernatant was collected to detect SOD, GSH, and MDA levels. All measurements were performed with an ELISA kit according to the kit instructions.

### Cell apoptosis assay

HSCs were added in 96-well plates and cultured in DMEM with 10% FBS for 24 h. HSCs were then treated with various reagents at indicated concentrations as described above. The morphology of apoptotic HSCs was evaluated using a Hoechst33258 staining kit according to the protocol. Photographs were taken under a fluorescence microscope (Olympus, Tokyo, Japan).

### Real-time polymerase chain reaction (PCR) analysis

HSCs were added in 96-well plates and cultured in DMEM with 10% FBS for 24 h. HSCs were then treated with various reagents at indicated concentrations as described above. Total RNA was extracted from HSCs using TRIzol reagent after digestion. The mRNA expressions of TGF-β, NF-κB, and iNOS were detected by real-time PCR. The RNA content was analysed by measuring the optical density at 260 nm. Prime Script RT Master Mix kit was used in cDNA synthesis. According to the protocol provided by the manufacturer, SYBR green premix was used in real-time PCR. Relative expression of target genes was normalized to actin. Results were given as a ratio compared with the vehicle control. The primer sequences used in this study are listed in Table 1.

**Table 1.** List of primers for real-time polymerase chain reaction (PCR).

Target	Full gene name	Primer	Sequence
TGF-β	transforming growth factor β	RP	tgctcgctttgtacaacagc
		FP	cccgaatgtctgacgtattg
NF-κB	nuclear factor kappa B	RP	gcatccaccatggaagacaa
		FP	gctaagtgtaggacactgtc
iNOS	inducible nitric oxide synthase	RP	gcttacctgggaatagcacc
		FP	cacggagaacagcagagttg

### Statistical analysis

All data were analysed using SPSS version 19 software. All results were expressed as mean ± standard deviation (SD). Significant differences were evaluated by one-way ANOVA followed by an LSD test, and *p* < 0.05 indicated a statistically significant result.

## Results

### Protective effects of AM complex on H_2_O_2_-induced oxidative injury in HSCs

**Figure 2.**
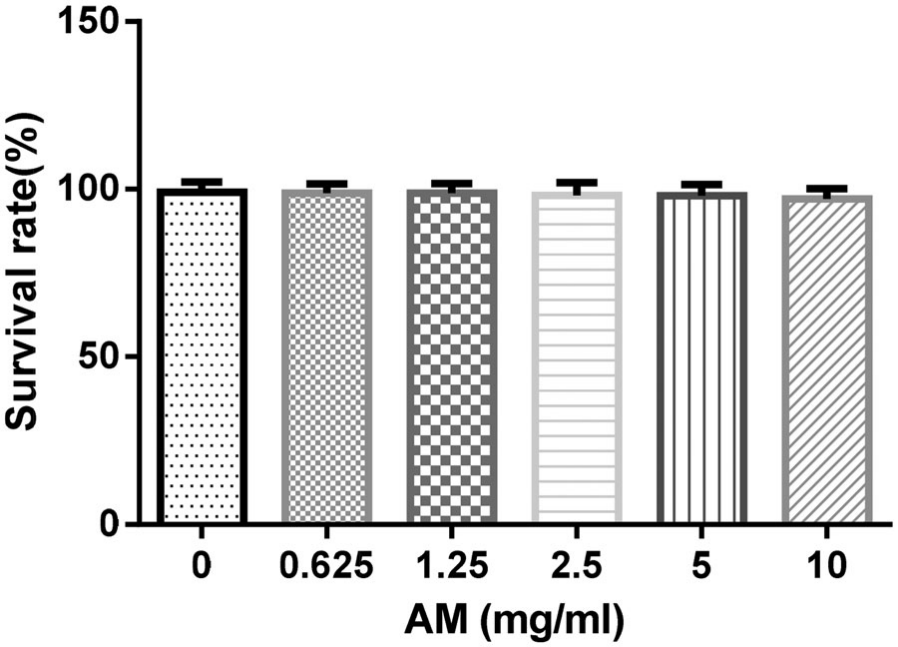
Toxicity of apigenin magnesium (AM) on hepatic stellate cells (HSCs). Values are presented as mean ± SD from three samples and were analysed using one-way ANOVA followed by an LSD test. There was no difference between groups.

**Figure 3.**
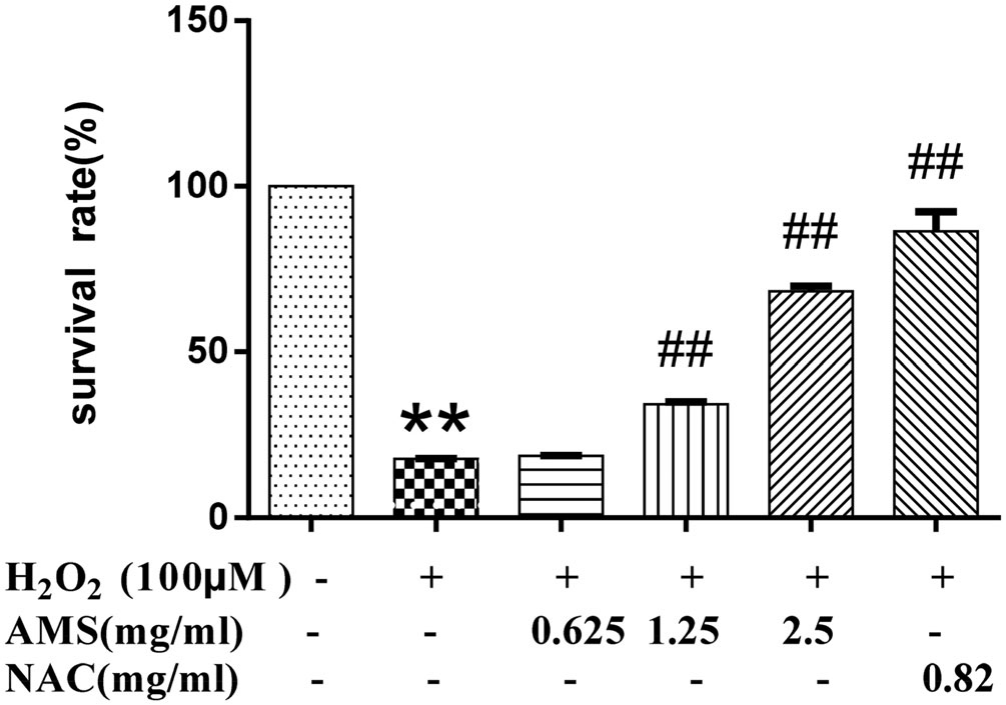
Effects of AM on HSC survival rate in the model of hydrogen peroxide (H_2_O_2_, 100 µM) induced oxidative stress. Values are presented as mean ± SD from three samples and were analysed using one-way ANOVA followed by an LSD test. *p < 0.05 and **p < 0.01 compared to the control group, ^#^*p* < 0.05 and ^##^*p* < 0.01 compared with the HSCs that received H_2_O_2_ alone.

The results showed that AM complex at concentrations up to 10.0 mg/mL did not significantly affect HSCs viability (IC_50_ = 1.56 × 10^5^ mg/mL), suggesting that AM complex might not be toxic to these cells (Figure 2). In addition, we found that H_2_O_2_ at 100 μM caused a significant reduction in HSC survival rate (*p* < 0.01). Accordingly, H_2_O_2_ of 100 μM was used to induce oxidative injury in HSCs for subsequent experiments. Next, we observed that AM complex increased cell survival rate in a concentration-dependent manner in HSCs exposed to H_2_O_2_, where concentrations of 1.25 and 2.5 mg/mL were statistically significant (*p* < 0.01) (Figure 3). In the H_2_O_2_-treated proliferation experiment, the IC_50_ of AM complex was 1.679 mg/mL. Overall, these data collectively indicated that AM complex protected HSCs against H_2_O_2_-induced oxidative injury.

### Antioxidative properties of AM complex in H_2_O_2_-treated HSCs

**Figure 4.**
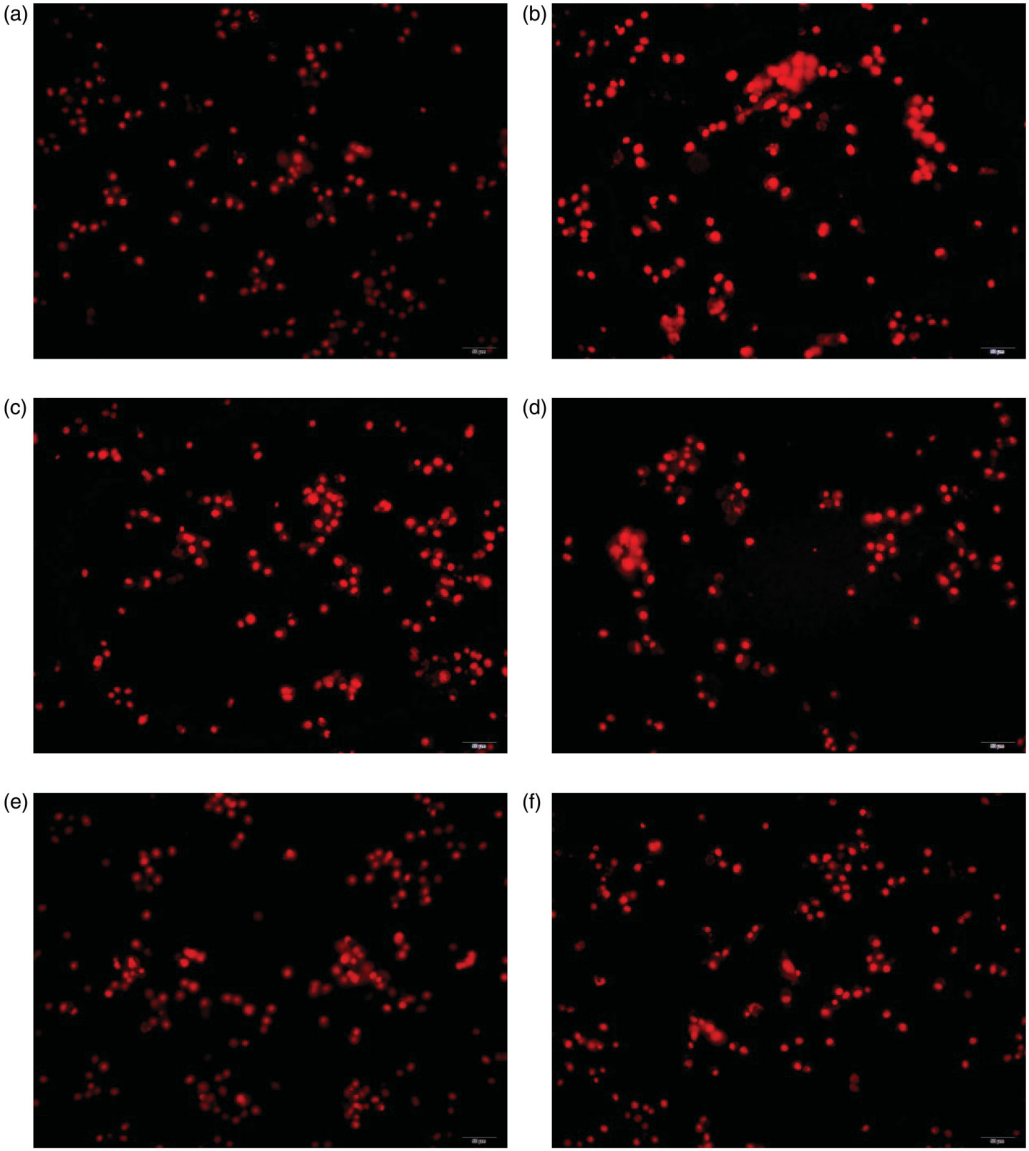
Effect of AM complex on intracellular superoxide levels after H_2_O_2_ exposure in HSC. (a) control; (b) model; (c) AM complex (0.625 mg/mL); (d) AM complex (1.25 mg/mL); (e) AM complex (2.5 mg/mL); (f) *N*-acetylcysteine (NAC, 0.82 mg/mL).

**Figure 5.**
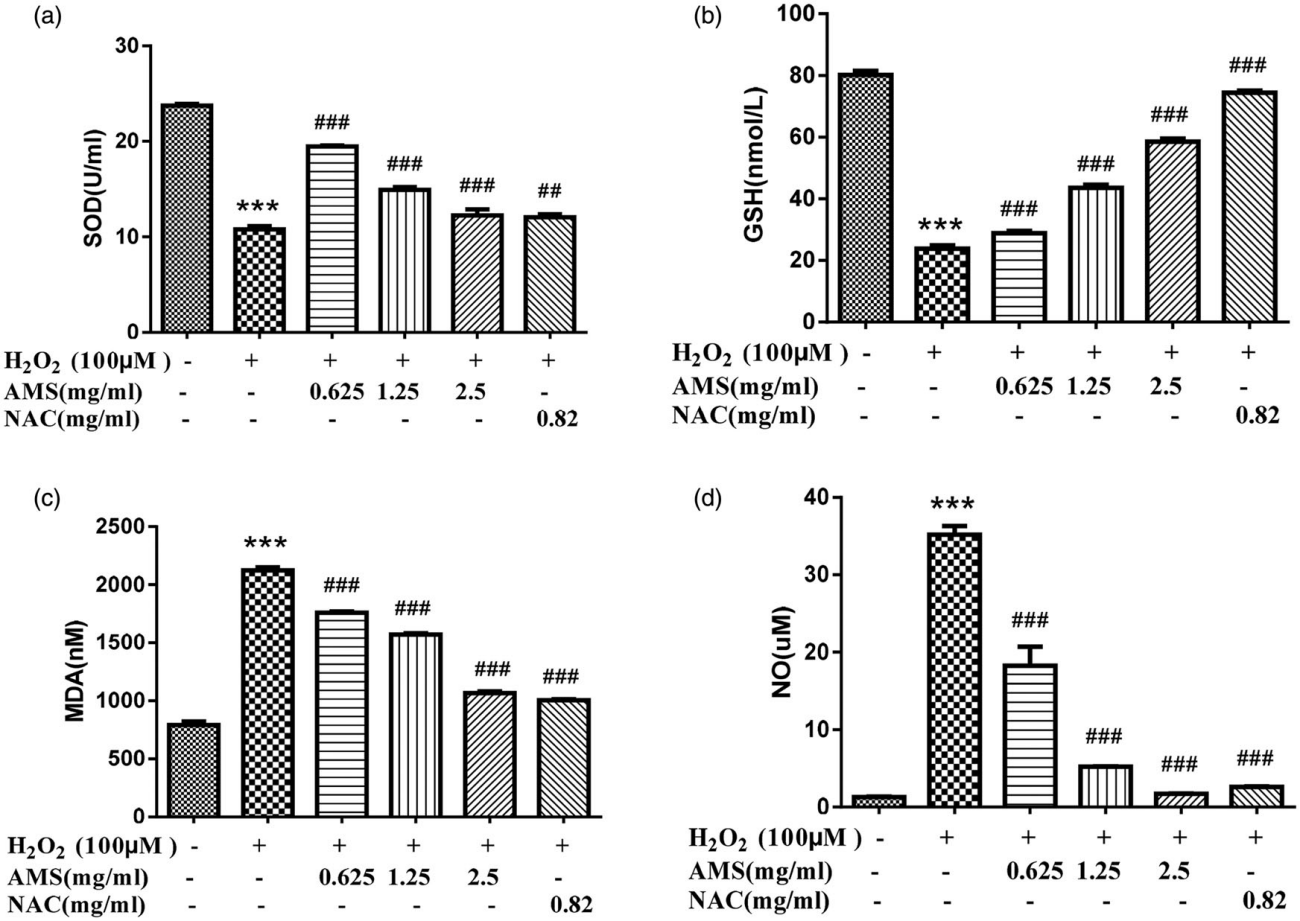
Effects of AM on superoxide dismutase (SOD), glutathione (GSH), malondialdehyde (MDA), and nitric oxide (NO) cellular levels in the model of H_2_O_2_ (100 µmol/L) induced oxidative stress. (a) SOD; (b) GSH; (c) MDA; (d) NO. Values are presented as mean ± SD from three samples and were analysed using one-way ANOVA followed by an LSD test. **p < 0.01 and ***p < 0.001 compared to the control group, ^##^*p* < 0.01 and ^###^*p* < 0.001 compared with the HSCs that received H_2_O_2_ alone.

We evaluated the protective effects of AM complex against oxidative stress damage in H_2_O_2_-treated HSCs. Fluorescence microscope analyses demonstrated that H_2_O_2_-treated HSCs had significantly high intracellular levels of ROS, but AM complex decreased ROS production (Figure 4). Additionally, we measured the classical phase II metabolizing enzyme activity of SOD in H_2_O_2_-treated HSCs. The results showed that SOD activity was significantly reduced in H_2_O_2_-treated HSCs, but AM complex of 0.625, 1.25, and 2.5 mg/mL all significantly restored SOD activity (*p* < 0.001, *p* < 0.001, and *p* < 0.001, respectively). However, the effects of AM complex on SOD were reduced as the concentration increased. Moreover, the levels of GSH, MDA, and NO, three parameters indicative of oxidative status within cells, were determined. As shown in Figure 5, HSCs challenged with 100 μM H_2_O_2_ exhibited significantly reduced GSH levels (*p* < 0.001) but significantly elevated MDA and NO levels (*p* < 0.001 and *p* < 0.001, respectively) compared with the control group levels. However, pretreating the cells with AM complex (0.625, 1.25, and 2.5 mg/mL) for 0.5 h restored GSH levels (*p* < 0.001) and decreased MDA and NO levels, which were previously significantly increased (*p* < 0.001 and *p* < 0.001, respectively). The effects of AM complex on GSH, MDA and NO levels were in concentration-dependent manners. Altogether, these results suggested that AM complex exerted great antioxidative properties in H_2_O_2_-treated HSCs.

### Inhibition of secretion of pro-inflammatory cytokines in HSCs

**Figure 6.**
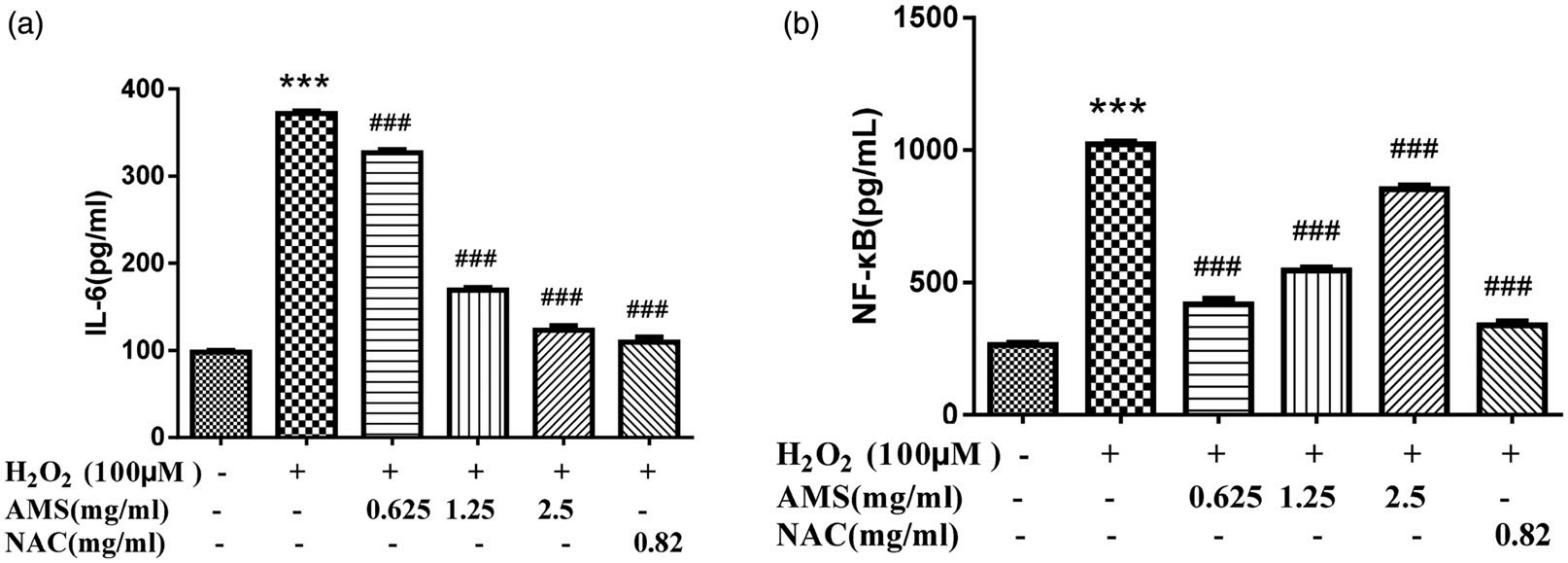
Effects of AM on interleukin 6 (IL-6) and nuclear factor-kappa B (NF-κB) cellular levels in the model of H_2_O_2_ (100 µmol/L) induced oxidative stress. (a) IL-6; (b) NF-κB. Values are presented as mean ± SD from three samples and were analysed using one-way ANOVA followed by an LSD test. **p < 0.01 and ***p < 0.001 compared to the control group, ^##^*p* < 0.01 and ^###^*p* < 0.001 compared with the HSCs that received H_2_O_2_ alone.

Changing levels of inflammatory factors IL-6 and NF-κB by pretreating with AM complex were determined in H_2_O_2_-stimulated HSCs (Figure 6). As expected, H_2_O_2_-treatment significantly increased IL-6 and NF-κB production. However, AM complex significantly inhibited IL-6 and NF-κB production at different concentrations (*p* < 0.001 and *p* < 0.001, respectively). Specifically, inhibitory effects of AM complex on IL-6 decreased with increasing AM complex concentration, with the strongest effect calculated at 0.625 mg/mL. In contrast, inhibitory effects on NF-κB increased with increasing AM complex concentration, with the strongest effect found at 2.5 mg/mL.

### Results of cell apoptosis

**Figure 7.**
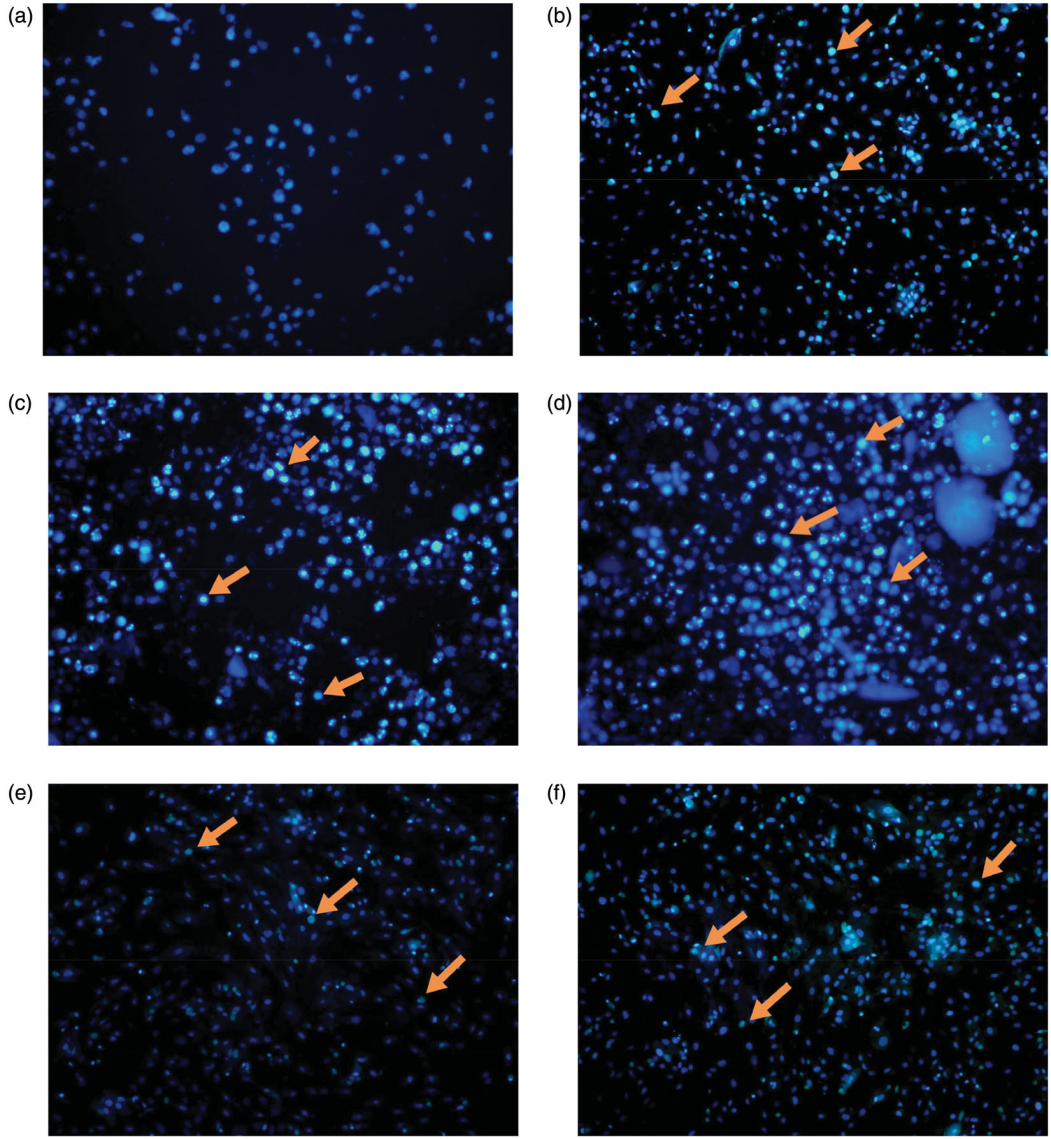
Effect of AM complex on apoptosis of HSCs after exposure to H_2_O_2_. The arrows indicate apoptotic cells. (a) control; (b) model; (c) AM complex (0.625 mg/mL); (d) AM complex (1.25 mg/mL); (e) AM complex (2.5 mg/mL); (f) NAC (0.82 mg/mL).

When the effects of AM complex on HSCs apoptosis were examined, our study indicated that HSCs treated with AM complex exhibited significant DNA condensation and fragmentation with brilliant blue staining (Figure 7). The effects on apoptosis rates are associated with concentration, with the strongest effects showing at 1.25 mg/mL.

### mRNA expressions of TGF-β, NF-κB, and iNOS

**Figure 8.**
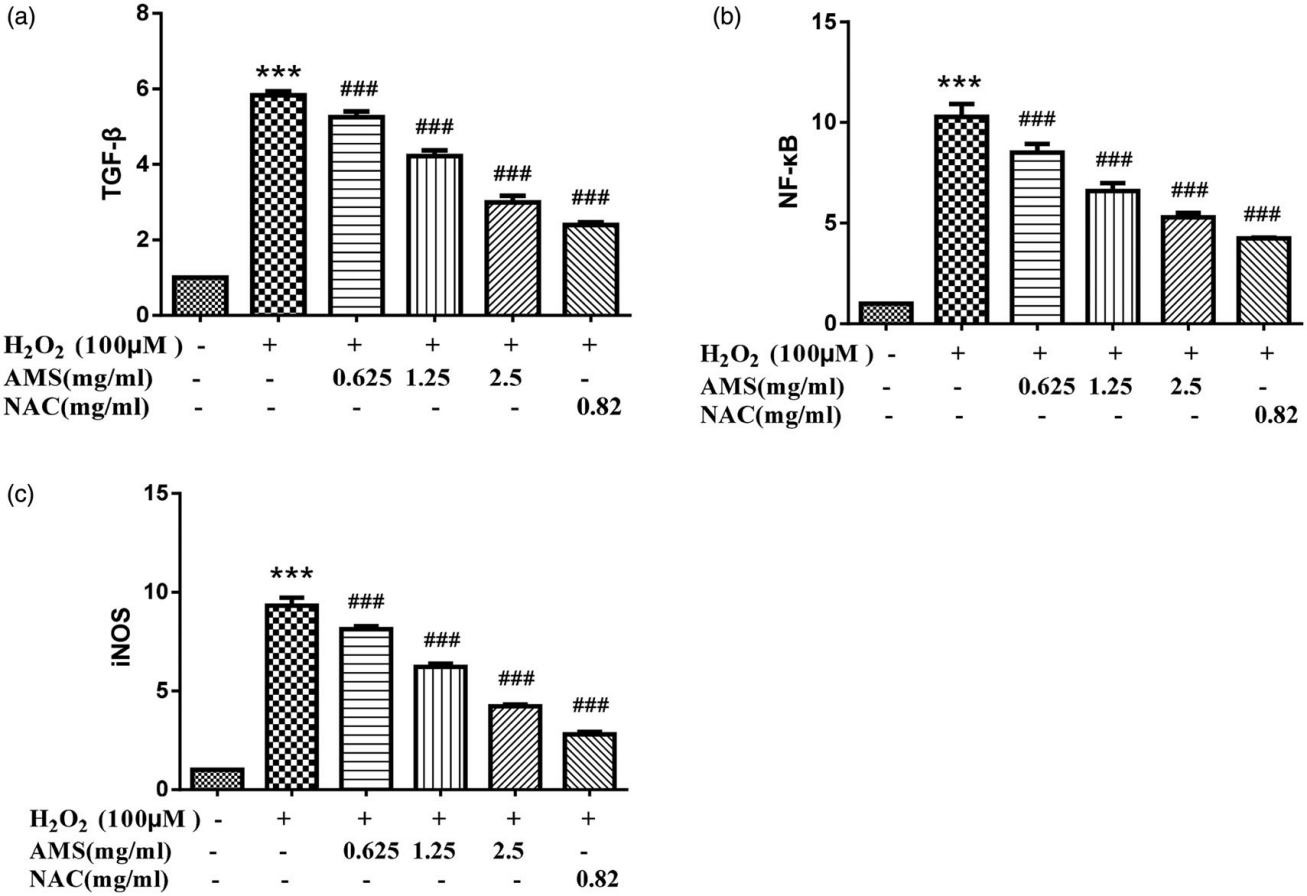
Effects of AM on mRNA expression of transforming growth factor-β (TGF-β), nuclear factor-kappa B (NF-κB), and inducible nitric oxide synthase (iNOS) in the model of H_2_O_2_ (100 µmol/L) induced oxidative stress. (a) TGF-β; (b) NF-κB; (c) iNOS. Values are presented as mean ± SD from three samples and were analysed using one-way ANOVA followed by an LSD test. **p < 0.01 and ***p < 0.001 compared to the control group, ^##^*p* < 0.01 and ^###^*p* < 0.001 compared with the HSCs that received H_2_O_2_ alone.

We next examined the roles of TGF-β, NF-κB, and iNOS signalling in AM complex protection of HSCs. Data indicated that AM complex could inhibit these pathways in H_2_O_2_-treated HSCs. As shown in Figure 8, mRNA expression of TGF-β, NF-κB, and iNOS increased in H_2_O_2_-treated HSCs (*p* < 0.001, *p* < 0.001, and *p* < 0.001, respectively) compared to the control’s mRNA expression. The increased mRNA expressions of TGF-β, NF-κB, and iNOS were reduced in a concentration dependent manner (*p* < 0.001, *p* < 0.001, and *p* < 0.001, respectively) in HSCs pre-treated with AM complex. Collectively, these data suggested that AM complex may inhibit TGF-β, NF-κB, and iNOS expression to protect against H_2_O_2_-induced inflammatory responses in HSCs.

## Discussion

Studies have indicated that apigenin exerts a protective effect against oxidative stress (Han et al. 2012; Huang et al. 2013). Apigenin has been shown to attenuate oxidative injury in ARPE-19 cells through activation of the Nrf2 pathway (Xu et al. 2016). Further studies show that apigenin inhibits lipid peroxidation and significantly increases the enzyme antioxidant defense mechanisms in paracetamol-induced hepatotoxicity in rats (Rašković et al. 2017). Additionally, many studies have demonstrated the anti-inflammatory activity of apigenin under a range of pathophysiological conditions. Apigenin promotes different anti-inflammatory pathways, including the p38/MAPK and PI3K/Akt pathways, as well as prevent IKB degradation and nuclear translocation of NF-κB, and reduce COX-2 activity (Huang et al. 2010). Moreover, apigenin may function as a potent anti-inflammatory and antifibrotic agent against bleomycin-induced lung fibrosis (Chen and Zhao 2016). These conclusions were consistent with our previous research (Xi et al. 2014; Shao et al. 2015; Zhuang et al. 2016). However, the effect of AM complex on oxidative stress and inflammatory response remained unknown.

In the present study, the protective effects of AM complex on oxidative stress and inflammatory responses were investigated on H_2_O_2_-induced rat HSCs. H_2_O_2_ exposure resulted in significant reductions in SOD activity and GSH levels and elevation of ROS, NO, MDA, IL-6, TGF-β, NF-κB, and iNOS levels in HSCs. However, AM complex treatment attenuated the reduction of SOD activity and GSH levels, and decreased ROS, NO, MDA, IL-6, TGF-βGFNF-κB, and iNOS levels in rat HSCs. Collectively, the present results suggest that AM complex exerts an obvious protective effect against H_2_O_2_-induced oxidative stress and inflammatory responses in HSCs.

Elucidation of the underlying effect of AM complex on oxidative stress and inflammatory responses is important for developing AM complex as a promising anti-hepatic disease candidate. Oxidative stress is an imbalance between ROS levels and antioxidant capacity in cells. ROS and reactive nitrogen species (RNS) are now being used to describe free radicals derived from molecular oxygen and oxidants derived from NO•, respectively (Turrens 2003; Zorov et al. 2014*)*. SOD is a superoxide scavenger and one of the most important protective enzymes, providing the first antioxidant defense system in various organs and tissues, including the liver. GSH is a key ROS scavenger to protect liver cells from oxidative stress, and GSH depletion in hepatocytes may endanger the antioxidant defense system leading to ROS accumulation. SOD and GSH play an important role in the antioxidant defense system, while MDA is considered to be a main marker of lipid peroxidation in tissues. In addition, all liver cells are capable of producing NO, including hepatocytes, hepatic macrophages, hepatic sinusoidal endothelial cells, and HSCs. NO is a free radical with high toxicity. Studies have shown that NO reacts with O_2_ to form superoxide nitrite anion ONOO−. ONOO − is a strong oxidant of protein and non-protein sulfhydryl groups, which is 50–1000 times stronger than NO. ONOO − attacks HSCs to initiate a chain reaction of lipid peroxidation. Therefore, NO amplifies the damage effect of O_2_ and is one of the main free radicals of oxidative stress injury (Thuy et al. 2017). As shown in Figure 5, AM complex inhibited the H_2_O_2_-induced increase of ROS through elevating SOD activity and GSH content, and decreasing MDA and NO levels. AM complex thus provides a protective effect against H_2_O_2_-induced oxidative stress. The increased SOD and decreased MDA effect of AM complex is better than apigenin (Pan et al. 2017), which is consistent with our assumption. It is possible that the formation of AM complex changes apigenin’s physicochemical property and increases its solubility.

Investigations focussing on the relationship and interaction of oxidative stress and inflammation have attracted great attention as accumulated evidence indicates that they are tightly correlated and orchestrated to drive the pathophysiological pathway of liver diseases (Biswas 2016; Gao and Tsukamoto 2016; Lam et al. 2016). Many ROS or RNS can augment proinflammatory gene expression by provoking an intracellular signalling cascade. In addition, inflammatory cells could produce more ROS/RNS, resulting in exaggerated oxidative stress as an inflammatory lesion. In the present investigation, H_2_O_2_-induced oxidative stress caused an inflammatory response by elevating IL-6 and NF-κB levels, and mRNA expression of TGF-β, NF-κB, and iNOS. The production of iNOS, one of the three NO synthetases inside the body, does not depend on the increase of Ca^2+^ concentration and is expressed only when the cells are stimulated by various cytokines, inflammation, and pathological immune responses and not under normal conditions. It has been reported that TGF-β, IL-6, and NF-κB can induce the mRNA expression of iNOS, thereby producing a large amount of NO (Chen et al. 2003; Vital et al. 2003). These were consistent with our findings in the H_2_O_2_-induced oxidative stress model. AM complex can also exhibit anti-inflammatory properties, which may be associated with its protective effect against oxidative stress.

## Conclusions

AM complex exerts an obvious protective effect on H_2_O_2_-induced oxidative stress and inflammatory response in rat HSCs. Hence, we suggest that AM complex may be developed as a promising hepatoprotective agent. Due to the good results found in this study, additional efforts both *in vivo* and in humans, are required to assess of AM complex as a potential therapeutic drug in liver disease.
